# Supplementary material for: Streptococcus pneumoniae triggers hierarchical autophagy through reprogramming of LAPosome-like vesicles via NDP52-delocalization
Source: Commun Biol. 2020 Jan 13;3:25. doi: 10.1038/s42003-020-0753-3 (PMC6957511; doi:10.1038/s42003-020-0753-3)
Supplement: Supplementary file 1 — Supplementary Information [file 42003_2020_753_MOESM1_ESM.pdf]

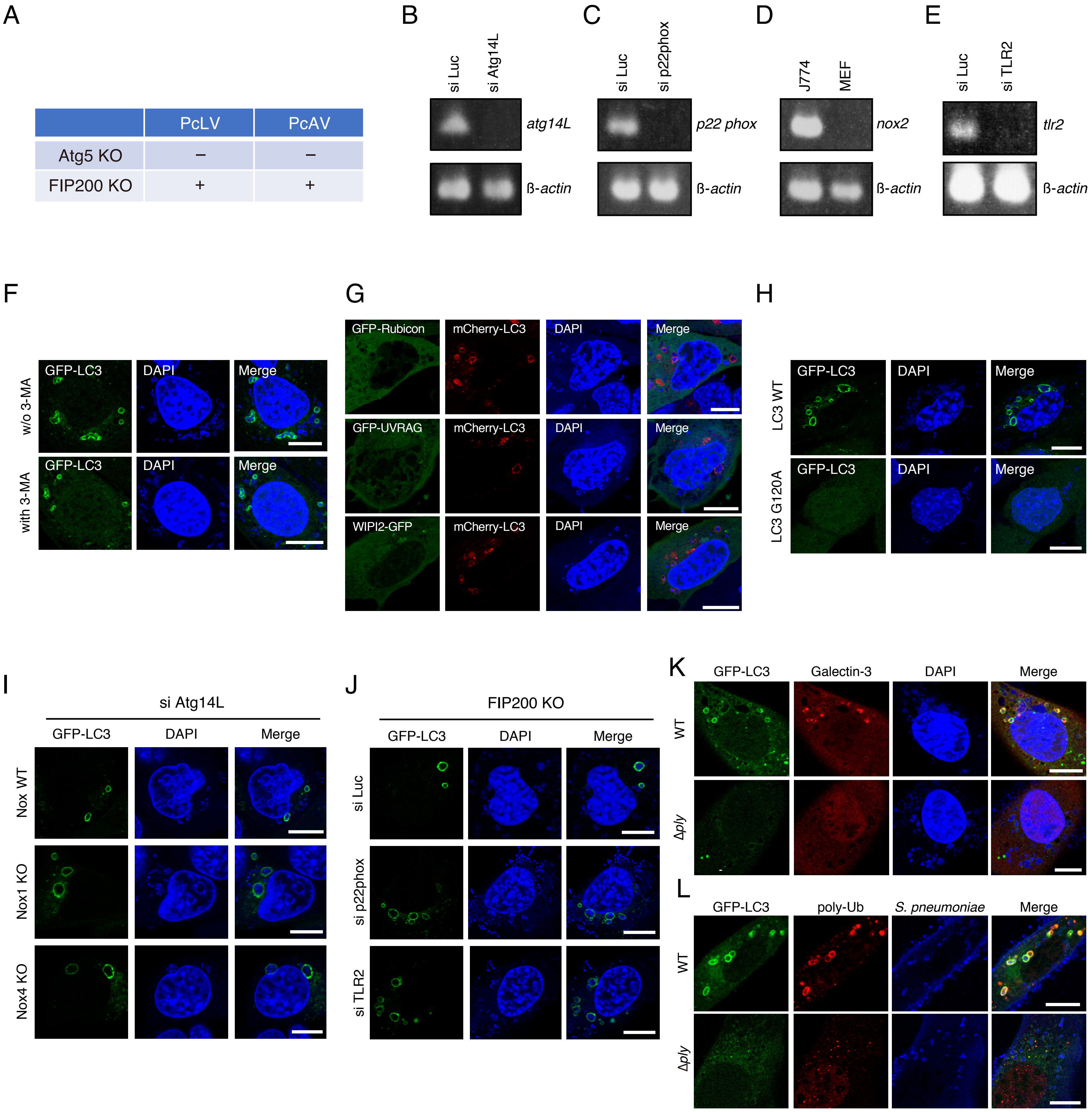

Supplementary Figure 1. Streptococcus pneumoniae is engulfed in FIP200-independent LAP-like vacuoles during early stage of infection

continued

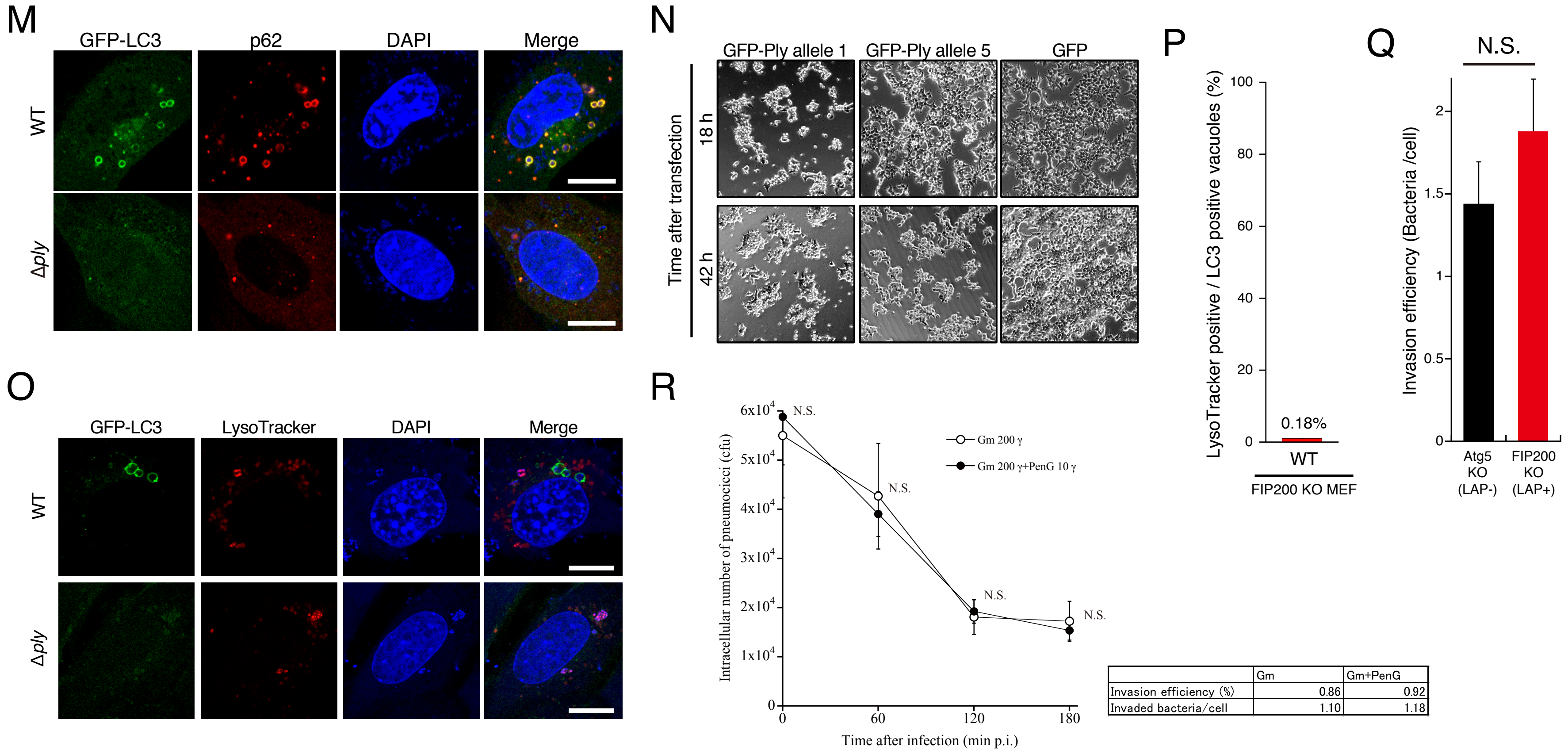

	Gm	Gm+PenG
Invasion efficiency (%)	0.86	0.92
Invaded bacteria/cell	1.10	1.18

Supplementary Figure 1. Streptococcus pneumoniae is engulfed in FIP200-independent LAP-like vacuoles during early stage of infection

Features of Atg5 KO and FIP200 KO MEFs in PcLV and PcAV formation capacity. (B-E) Knockdown effect of indicated specific mouse siRNA was evaluated by RT-PCR using total mRNA from each cell as a template and visualized by agarose-gel-electrophoresis. (F) FIP200 KO MEFs/GFP-LC3 infected with pneumococci for 1 h with or without 3-methyladenine were stained with DAPI. (G) FIP200 KO MEFs/mChery-LC3 transiently expressing indicated host factors were infected with pneumococci for 1 h and stained with DAPI. (H) FIP200 KO MEFs/GFP-LC3 G120A infected with pneumococci for 1 h were stained with DAPI. (I and J) Indicated MEFs/GFP-LC3 treated with indicated siRNA were infected with pneumococci for 1 h and stained with DAPI. (K) FIP200 KO MEFs/GFP-LC3 infected with WT or Δply pneumococci for 1 h were stained with DAPI and anti-Galectin-3 antibody. (L and M) FIP200 KO MEFs/GFP-LC3 infected with WT or Δply pneumococci for 1 h were stained with DAPI or antibodies against pneumococci, and anti-poly-Ub or -p62 antibodies. (N) Phase-contrast images of 293T cells transiently expressing indicated protein for 18 or 42 h. Magnification: x20. (O) FIP200 KO MEFs infected with WT or Δply pneumococci for 1 h in the presence of 50 nM LysoTracker were stained with DAPI. (P) Percentage of LysoTracker-positive PcLVs in WT bacteria infected cells in (O). (Q) Atg5 KO or FIP200 KO MEFs were infected with pneumococci and invasion efficiency of bacteria was determined by colony forming units (cfu); n = 3. (R) WT MEFs were infected with pneumococci and extracellular bacterium was killed by 30 min treatment of 200 μg/ml gentamycin or {(15 min treatment of 200 μg/ml gentamycin) + (15 min treatment of 200 μg/ml gentamycin+10 μg/ml penicillin G)}, and intracellular survivability of bacteria was determined by colony forming units (cfu); n = 3. Data are expressed as mean ±SEM; N.S., not significant. Bar, 10 μm. Gm: gentamycin, PenG: penicillin G.

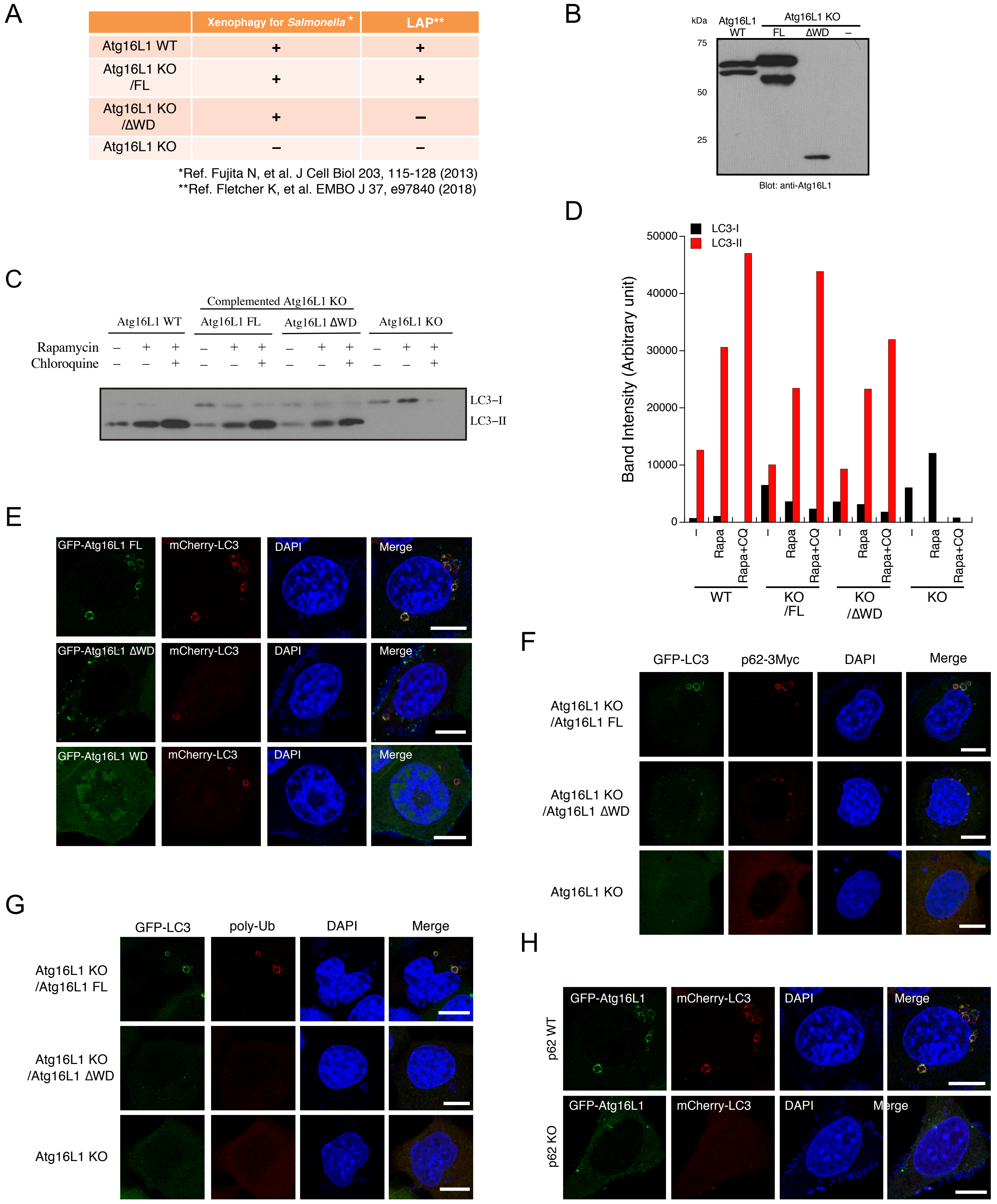

Supplementary Figure 2. Characterization of complemented Atg16L1 KO MEFs

(A) Reported features of Atg16L1 KO MEFs stably complemented with variety of Atg16L1 constructs. (B) Western blot analysis of Atg16L1 in Atg16L1 KO MEFs stably re-expressing indicated Atg16L1 constructs. (C) Western blotting of LC3 in Atg16L1 FL-, ΔWD-, or WD-complemented Atg16L1 KO MEFs with or without rapamycin and chloroquine. (D) Quantification of band intensity in (C). (E) Atg16L1 KO/MEFs/mCherry-LC3 transiently expressing GFP-Atg16L1 FL, ΔWD, or WD infected with pneumococci for 1 h were stained with DAPI. (F and G) Complemented Atg16L1 KO MEFs/GFP-LC3 transiently expressing p62-3Myc were infected with pneumococci for 1 h and stained with DAPI and indicated antibodies. (H) p62 WT or KO MEFs/mCherry-LC3 transiently expressing GFP-Atg16L1 infected with pneumococci for 1 h were stained with DAPI. Bar, 10 μm.

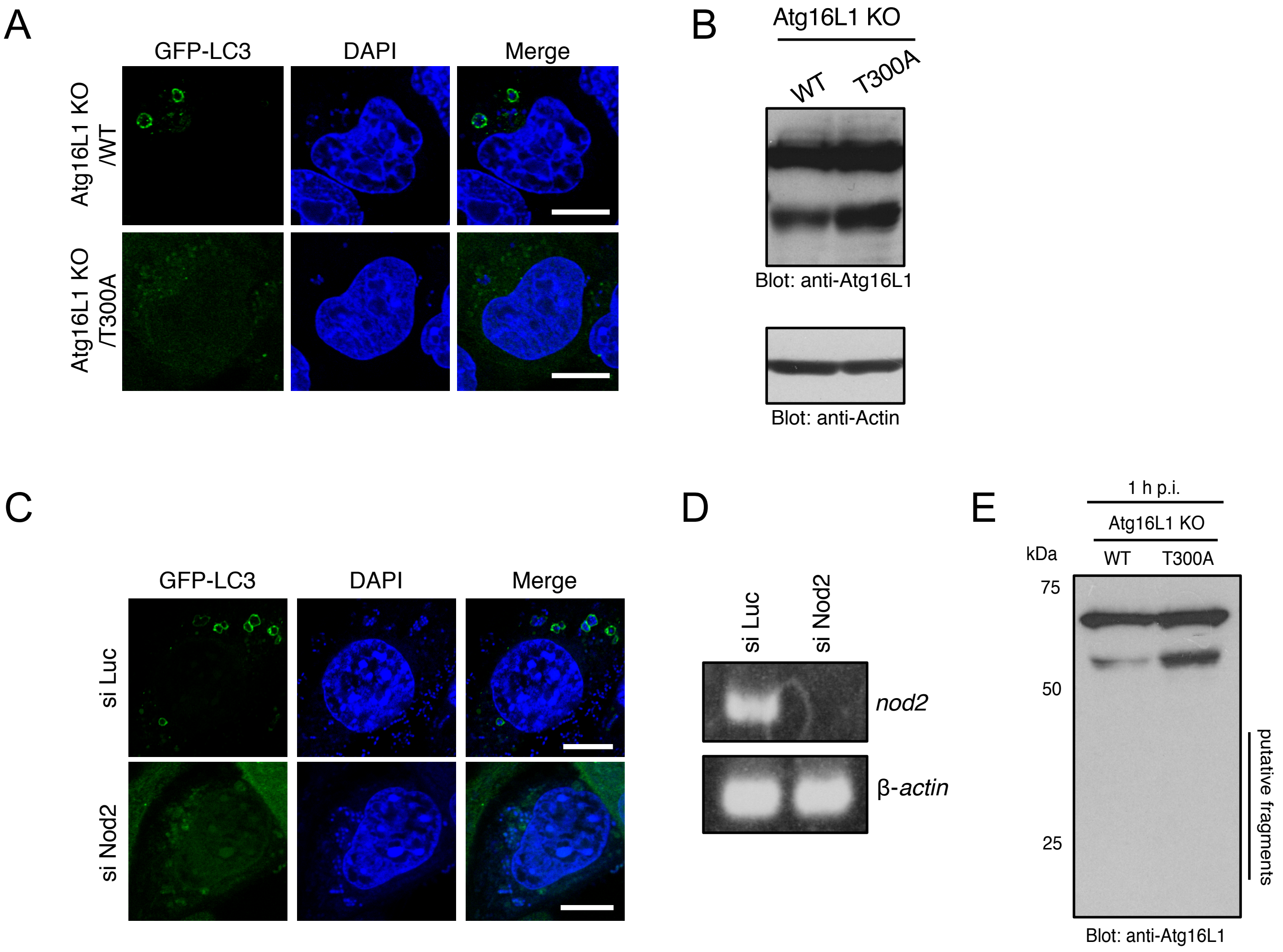

Supplementary Figure 3. Atg16L1 WD domain and T300 is involved in PcLV formation

(A) Complemented Atg16L1 KO MEFs /GFP-LC3 treated with siAtg14L were infected with pneumococci for 1 h. (B) Western blot analysis of Atg16L1 in Atg16L1 KO MEFs stably re-expressing indicated Atg16L1 constructs. (C) FIP200 KO MEFs/GFP-LC3 treated with indicated siRNA were infected with pneumococci for 1 h and stained with DAPI. (D) Knockdown effect of mouse NOD2 specific siRNA was evaluated by RT-PCR using total mRNA from siRNA treated cells as a template and visualized by agarose-gel-electrophoresis. (E) Lysates of complemented Atg16L1 KO MEFs infected with pneumococci for at 1 h were analyzed by western blotting. Bar, 10 µm.

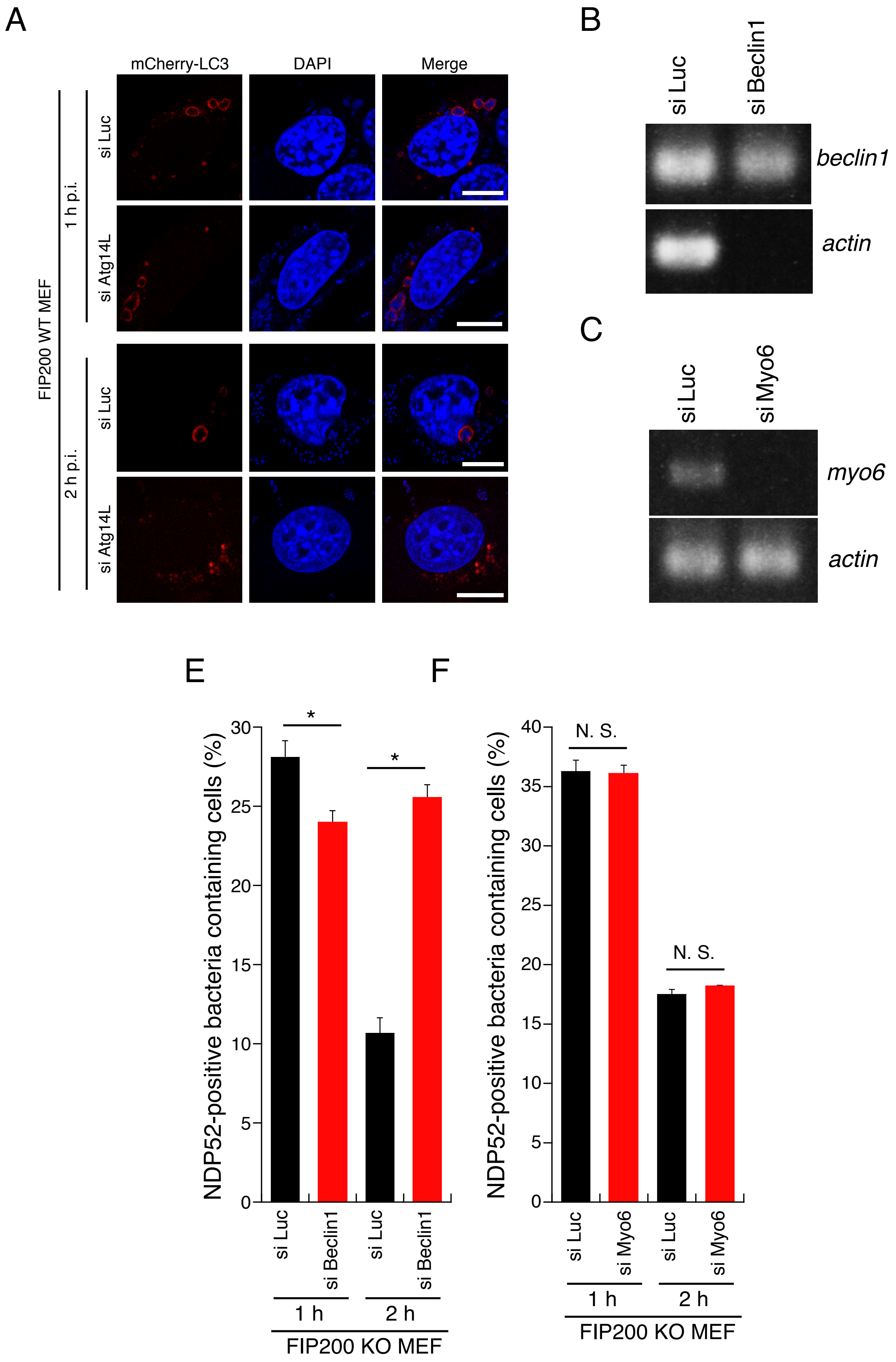

Supplementary Figure 4. Analysis of NDP52-delocalized PcLV transition

(A) FIP200 WT MEFs/mCherry-LC3 treated with indicated siRNA were infected with pneumococci for 1 or 2 h and stained with DAPI. (B and C) Knockdown effect of indicated specific mouse siRNA was evaluated by RT-PCR using total mRNA from each cell as a template and visualized by agarose-gel-electrophoresis. (D and E) FIP200 KO MEFs/mCherry-LC3/GFP-NDP52 treated with indicated siRNA were infected with pneumococci for 1 or 2 h and stained with DAPI, and NDP52-positive bacteria containing cells were quantified. Data are expressed as mean ± SEM.; *P < 0.01, N.S., not significant. Bar, 10 μm.

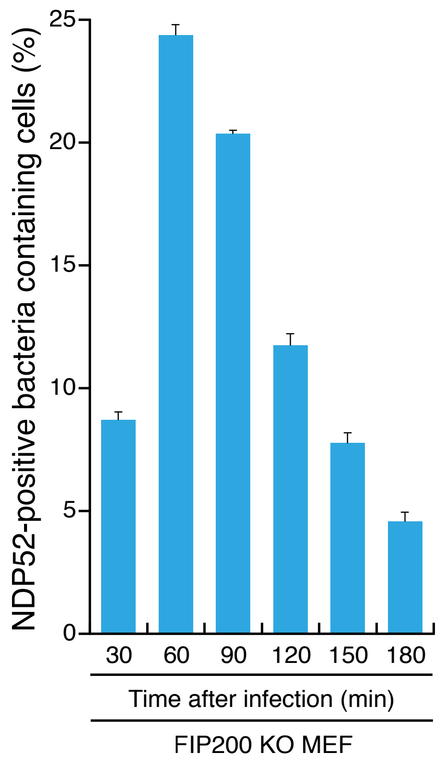

Supplementary Figure 5. Hierarchical autophagy processes during time course of pneumococcal infection

FIP200 KO MEFs/GFP-NDP52 infected with pneumococci for indicated periods were stained with DAPI, and percentages of GFP-NDP52 positive bacteria containing cells were quantified.

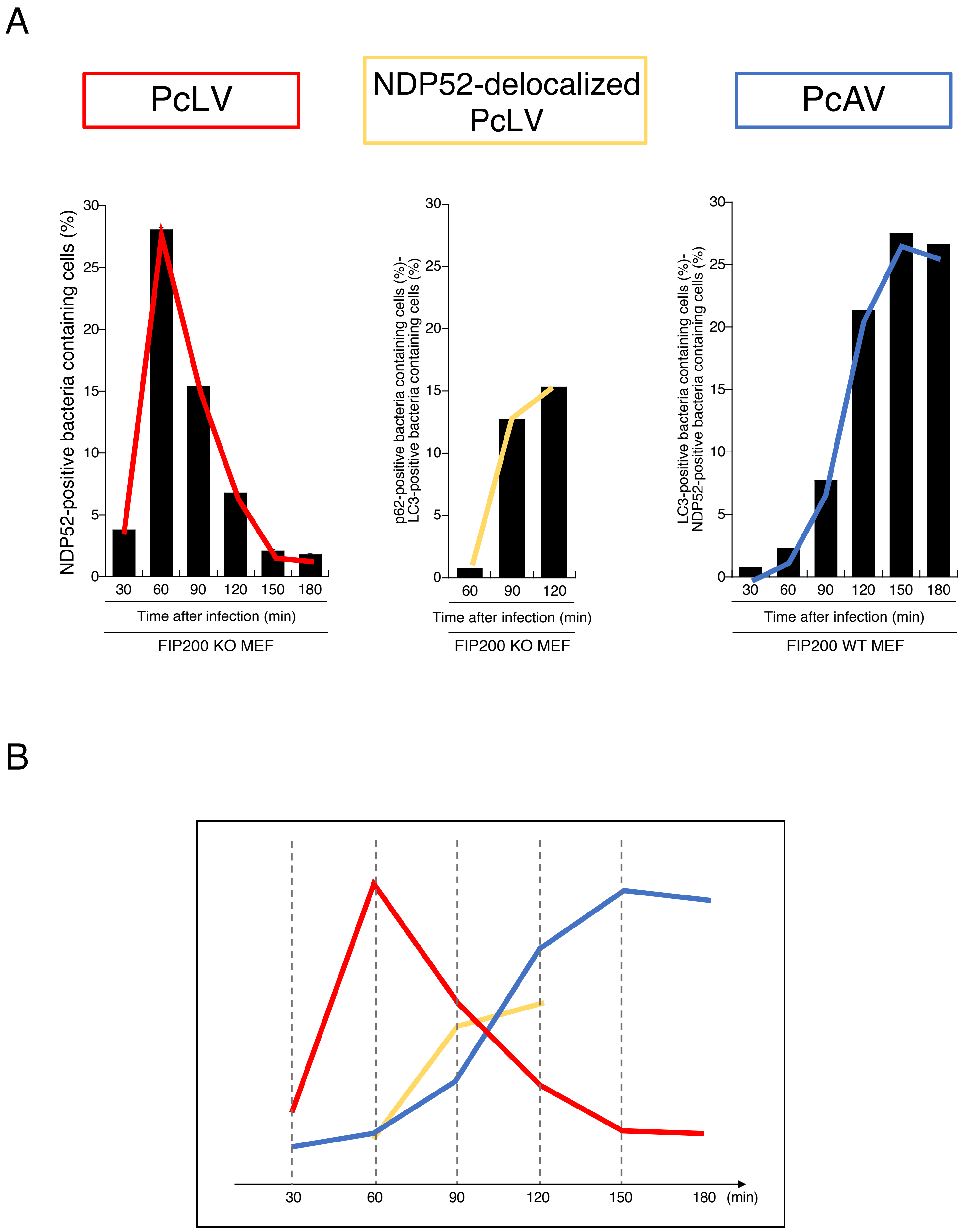

Supplementary Figure 6. Schematic models of hierarchical autophagy processes during time course of S. pneumoniae infection

(A) PcLV dynamics was depicted based on the population of NDP52 positive cells in Fig 4A. NDP52-delocalized PcLV dynamics was depicted based on the (population of p62 positive cells) - (population of LC3 positive cells) in Fig 4C. PcAV dynamics was depicted based on the (population of LC3 positive cells) - (population of NDP52 positive cells) in Fig 4A. (B) Schematic models of hierarchical autophagy processes during time course of S. pneumoniae infection.

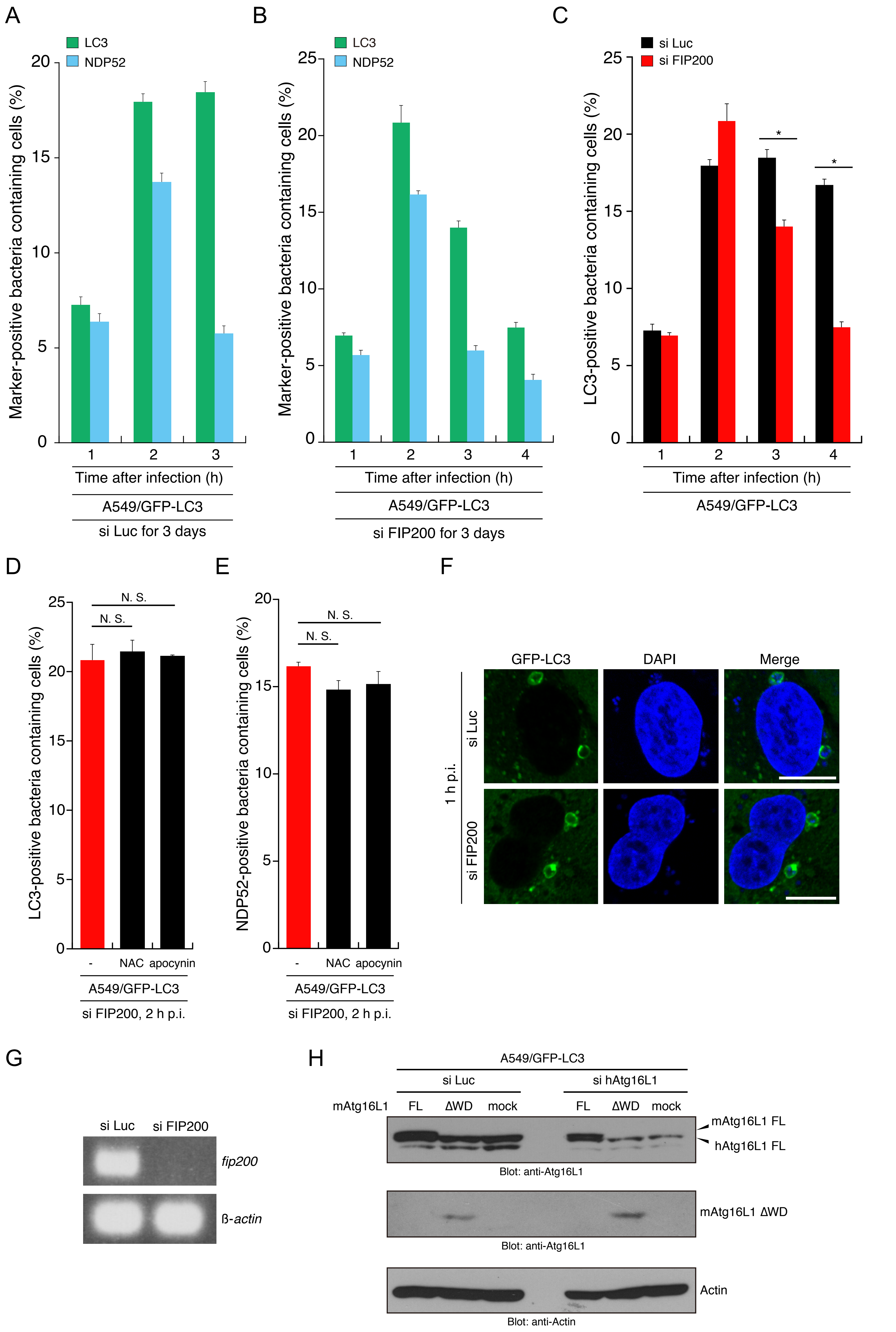

Supplementary Figure 7. FIP200- and ROS-independent transient PcLV formation in human pulmonary epithelial cells

continued

Supplementary Figure 7. FIP200- and ROS-independent transient PcLV formation in human pulmonary epithelial cells

(A and B) A549 cells/GFP-LC3 treated with indicated siRNA for 3 days were infected with pneumococci for indicated periods, and stained by anti-NDP52 antibody and DAPI, and percentages of each marker-positive bacteria containing cells were quantified. (C) Quantification results of LC3-positive bacteria containing cells in (A) and (B) are summarized in one plot. (D and E) A549 cells/GFP-LC3 treated with siFIP200 for 3 days were infected with pneumococci for 2 h in the presence or absence of 10 mM NAC and 300 μM apocynin, and stained by anti-NDP52 antibody and DAPI, and percentages of each marker-positive bacteria containing cells were quantified. (F) A549 cells/GFP-LC3 treated with indicated siRNA were infected with pneumococci for 1 h and stained with DAPI. (G) Knockdown effect of indicated specific mouse siRNA was evaluated by RT-PCR using total mRNA from each cell as a template and visualized by agarose-gel-electrophoresis. (H) Western blot analysis of Atg16L1 or actin in indicated siRNA treated A549 cells stably expressing siRNA-resistant mouse Atg16L1 FL, ΔWD, or mock. Data are expressed as mean ± SEM.; *P < 0.01, N.S., not significant. Bar, 10 μm.

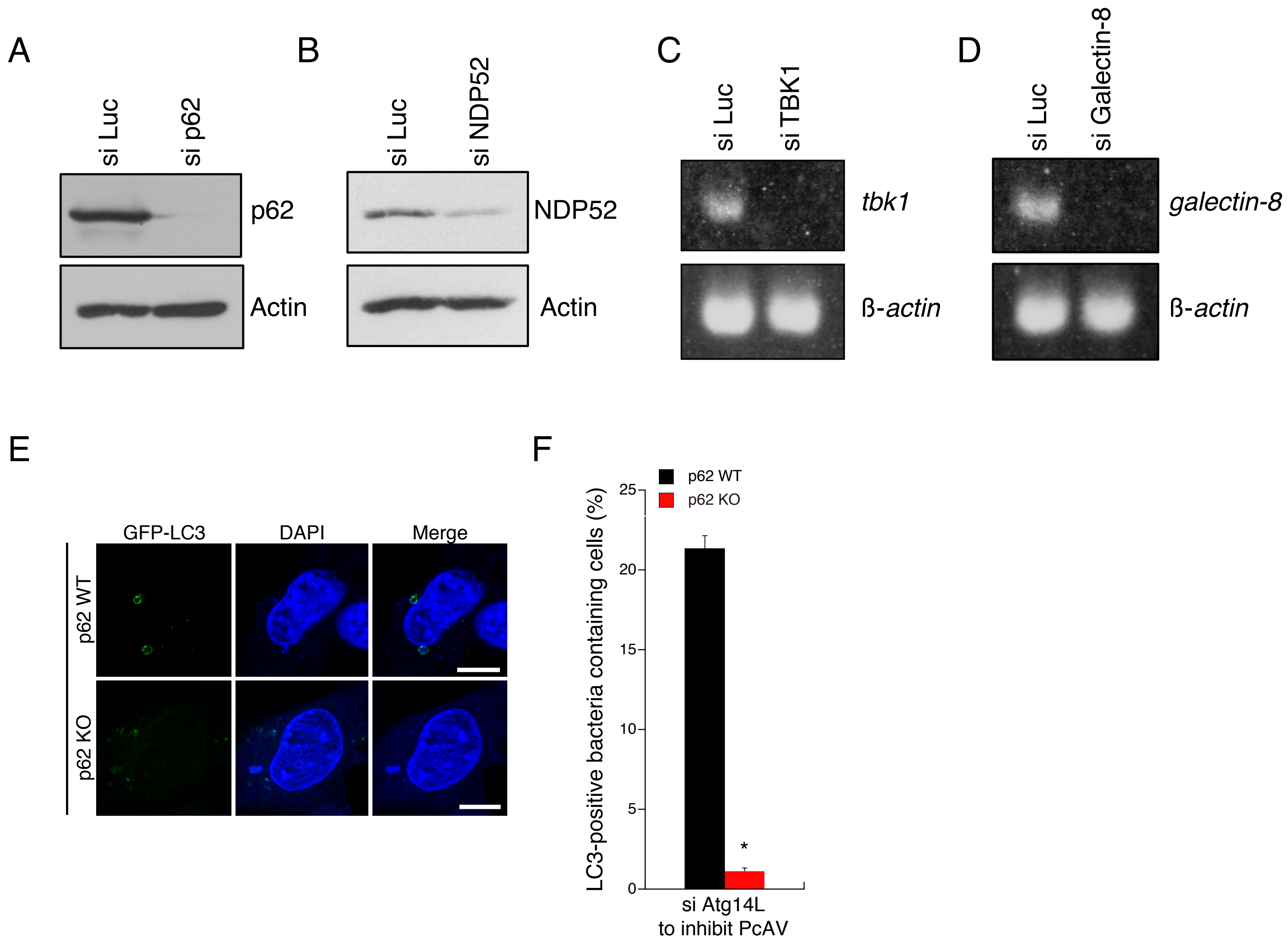

Supplementary Figure 8. Poly-Ub, p62 and NDP52 regulate PcLV formation interdependently

(A and B) Knockdown effect of indicated specific mouse siRNA was evaluated by western blotting using total cell lysates and indicated antibodies. (C and D) Knockdown effect of indicated specific mouse siRNA was evaluated by RT-PCR using total mRNA from each cell as a template and visualized by agarose-gel-electrophoresis. (E) p62 WT or KO MEFs/GFP-LC3 treated with siAtg14L were infected with pneumococci for 1 h and stained by DAPI. (F) Percentages of LC3-positive bacteria containing cells in (E) were quantified. Data are expressed as mean ± SEM.; *P < 0.01. Bar, 10 µm.

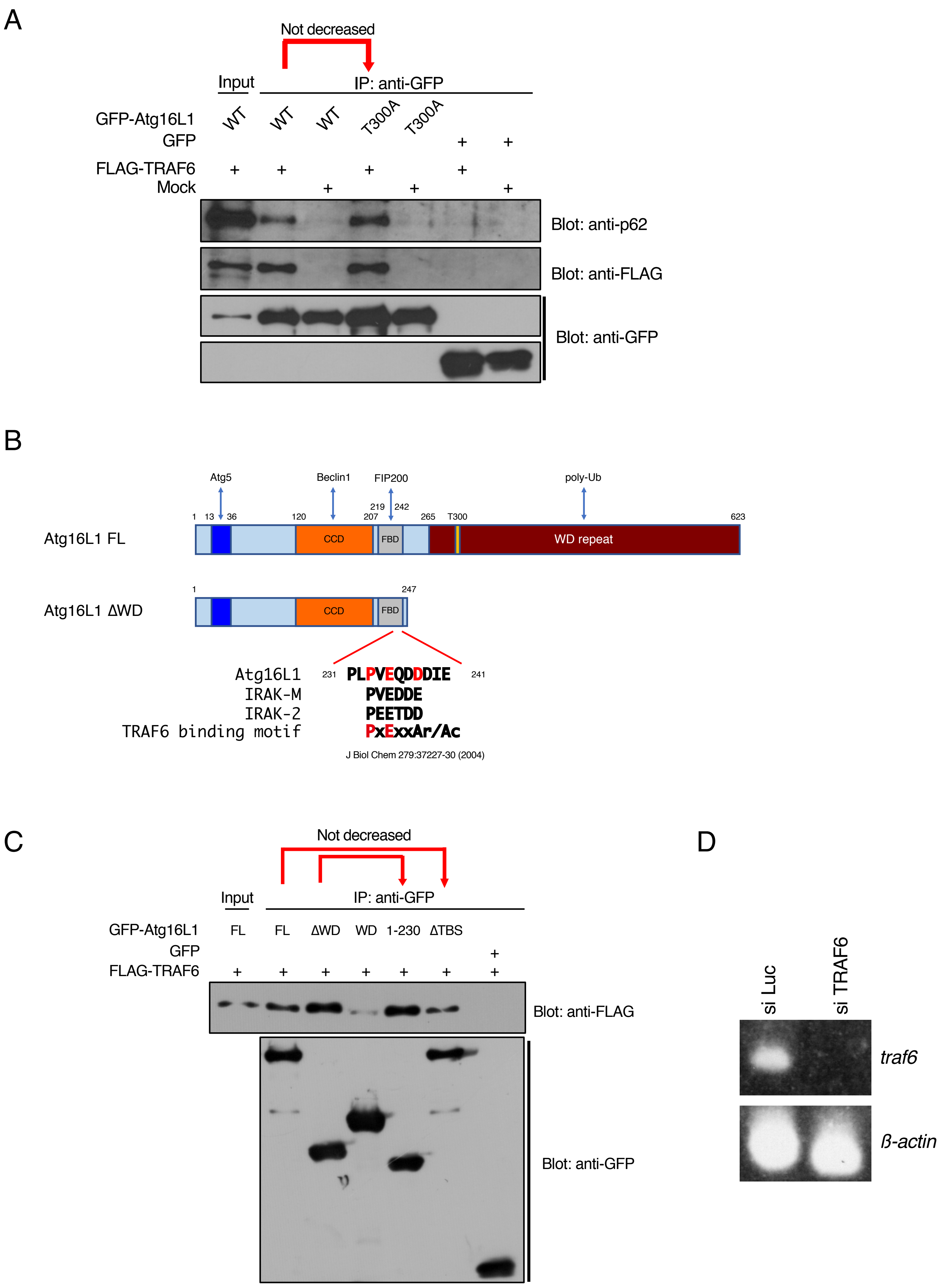

Supplementary Figure 9. Domain analysis of Atg16L1 in TRAF6-binding and knockdown effect of specific siRNA for TRAF6

(A) Lysates of 293T transiently expressing GFP-Atg16L1 variants and FLAG-TRAF6 were immunoprecipitated with GFP-Trap, and the bound proteins were immunoblotted with indicated antibodies. (B) Schematic Diagram of putative TRAF6 binding motif in Atg16L1. (C) Lysates of 293T transiently expressing GFP-Atg16L1 variants and FLAG-TRAF6 were immunoprecipitated with GFP-Trap, and the bound proteins were immunoblotted with indicated antibodies. (D) Knockdown effect of mouse TRAF6 specific siRNA was evaluated by RT-PCR using total mRNA from siRNA treated cells as a template and visualized by agarose-gel-electrophoresis.

Fig 1J

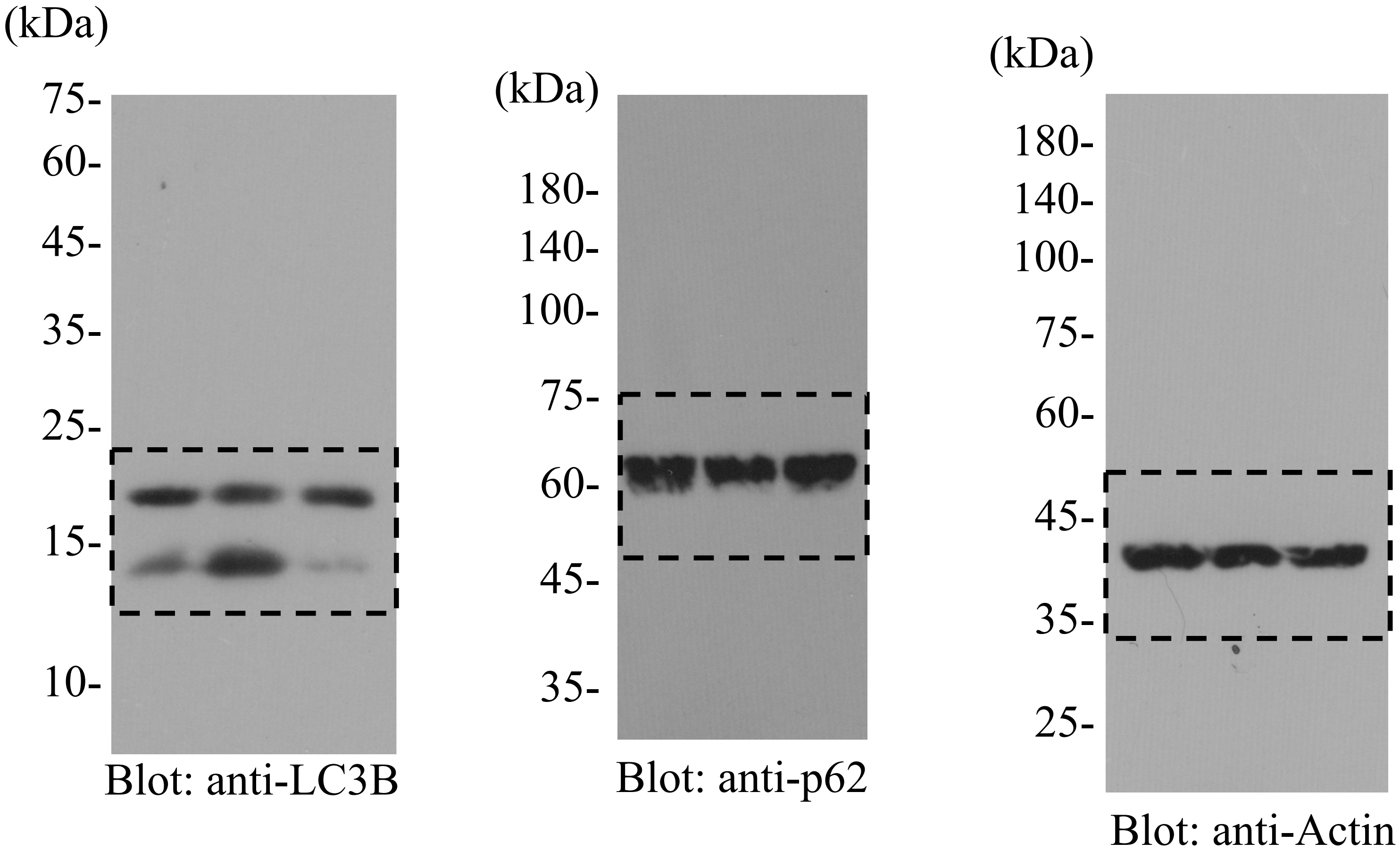

Supplementary Figure 10. Uncropped blots for Fig. 1J.

Fig 6A

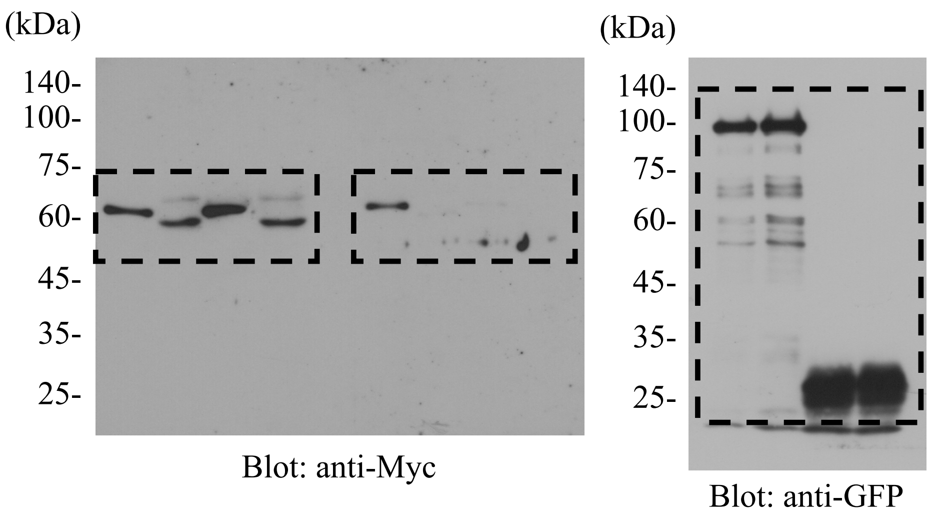

Fig 6C

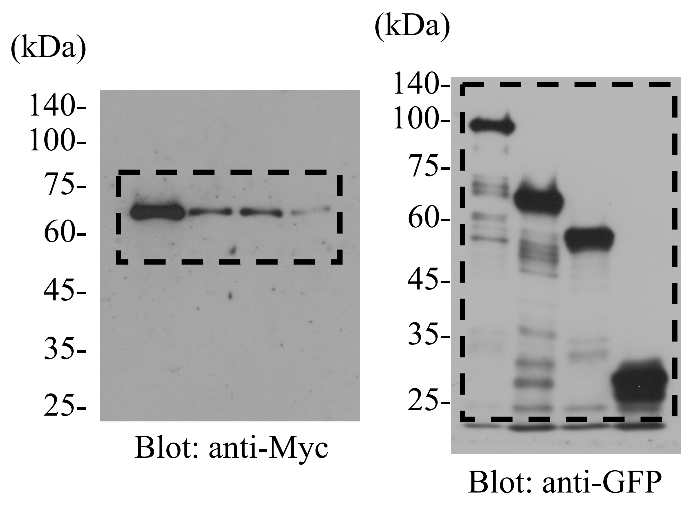

Fig 6E

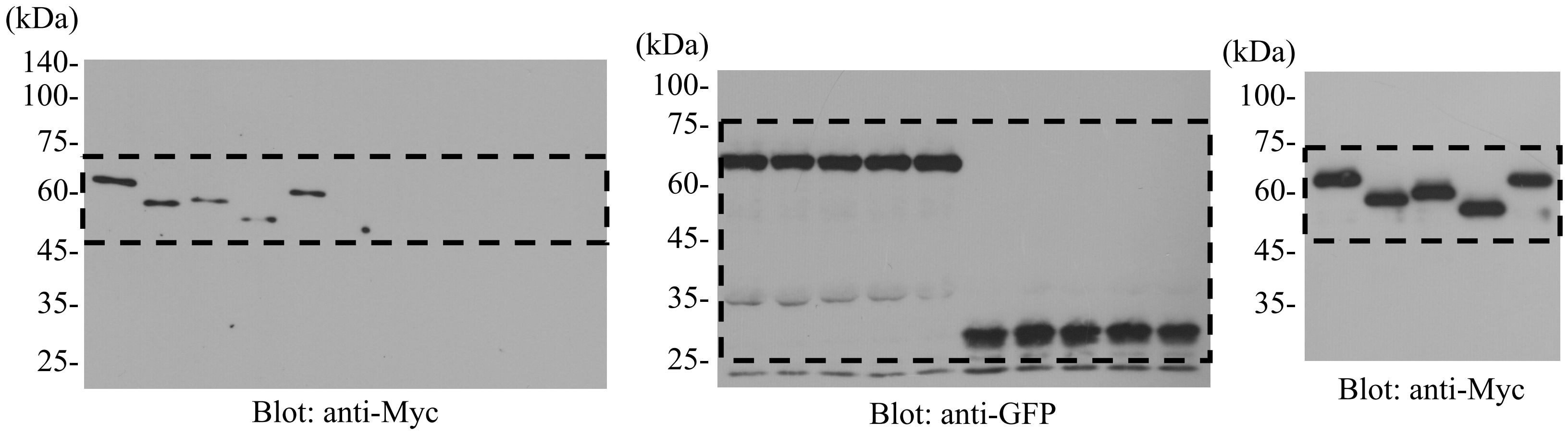

Fig 6F

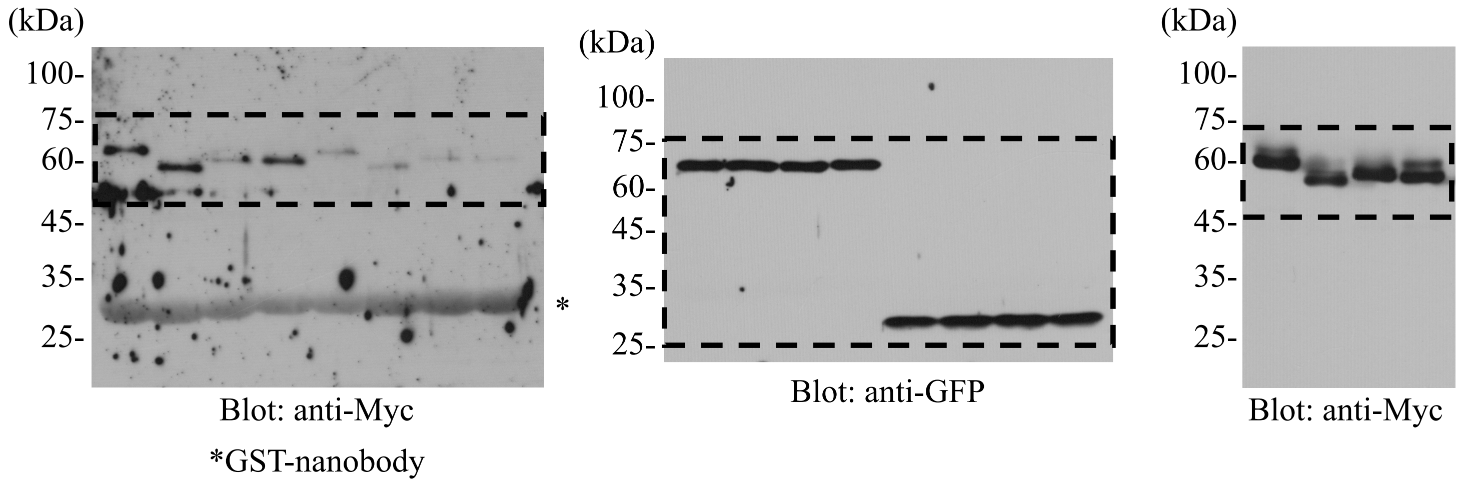

Fig 6G

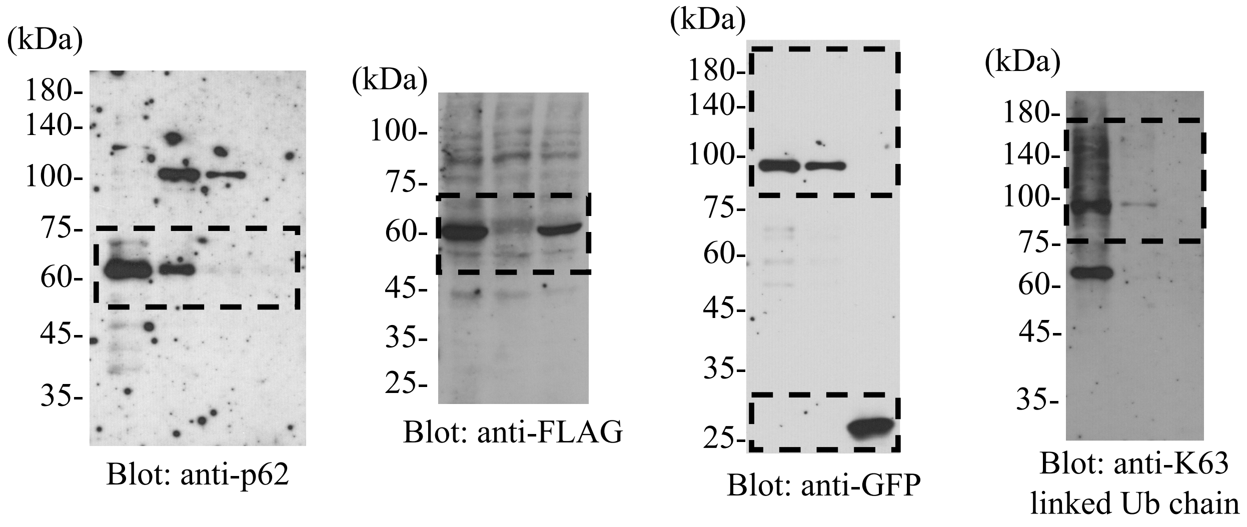

Supplementary Figure 11. Uncropped blots for Fig. 6A, C, E, F, and G.

Supplementary Table 1. Sequences of Expression Vector Cloning Primers.

human *uvrag*-F	GGAAGATCTATGAGCGCCTCCGCGTCGG
human *uvrag*-R	GGCCGACGTCGACTCACTTATCGGAACTCCTGC
human *optn*-F + kozac	CGCGGATCCGCCGCCACCATGGCTATGTCCCATCAACCTCTCAG
human *optn*-R	GGCCGACGTCGACAATGATGCAATCCATCACGTG
human *ndp52*-F + kozac	CGCGGATCCGCCGCCACCATGGAGGAGACCATCAAAGATCCC
human *ndp52*-R	GGCCGACGTCGACGAGAGAGTGGCAGAACACGTGGTC
mouse *atg16L1*-F	CCCAAGCTTATGTCGTCGGGCCTGCGC
mouse *atg16L1*-F + kozac	CCTTAATTAACGCCACCATGTCGTCGGGCCTGCGC
mouse *atg16L1*-R	GGCCGACGTCGACTCAAGGCTGTGCCCACAGC
mouse *atg16L1*ΔWD-R	GGCCGACGTCGACTTAGGTCTCTTCTGTGTGGTCTG
mouse *atg16L1* WD-F	GGAAGATCTTCAGACCACACAGAAGAGACC
human *p62* ΔZZ-F	CCCGCAACTTCCCCAGCCCCTTCGGG
human *p62* ΔZZ-R	GCTCAGGAGGCGCCCCGCAACTTCCCCA
human *p62* ΔTBS-F	CTTCTGGTGTTGATATCGATGTGGAGCACGGA
human *p62* ΔTBS-R	TATCAACACCAGAAGCTGATTCTGCCGT
human *p62* ΔKIR-F	TGTCTTCACAGATGCCAGAATCCGAAG
human *p62* ΔKIR-R	GCATCTGTGAAGACAGATGGGTCCAGTC
rat *lc3B* G120A-F	GCCTCCCAGGAGACGTTCGCGACAGCACTGGCTGTTACATAC
rat *lc3B* G120A-R	GTATGTAACAGCCAGTGCTGTCGCGAACGTCTCCTGGGAGGC
mouse *atg16L1* T300A-F	CCAGGATATCATGGACGCTCATCCTGCTTCTGG
mouse *atg16L1* T300A-R	CCAGAAGCAGGATGAGCGTCCATGATATCCTGG
ply R6 F-*Bam*HI	CGCGGATCCATGGCAAATAAAGCAGTAAATGAC
ply R6 R-*Xho*I	CCGCTCGAGCTAGTCATTTTCTACCTTATC
ply Allele5 F-*Bam*HI	CGCGGATCCATGGCAAATAAAGCAGTAAATGAC
ply Allele5 R-*Xho*I	CCGCTCGAGCTAATCATTTTCTACCTTATCCTC
mouse *atg16L1*-R (1-230aa)	GGCCGACGTCGACTTATTCCTTTGCTGCTTCTGCAAGC
mouse *atg16L1* ΔTBS-F	CAAAGGAAGACATTGAAGTCATTGTGGATGAG
mouse *atg16L1* ΔTBS-R	CAATGTCTTCCTTTGCTGCTTCTGC

Supplementary Table 2. Design of the siRNA for Knockdown Experiment, and Primer Sequences for Detecting Gene Expression by RT-PCR.

siAtg14	CAGGUGGUGGGGUUAACUAGUAG
sip22phox	UCCCAUUGAGCCUAAACCCAAGG
siTLR2	UCCUGCGAACUCCUAUCCUUUAC
sip62	UGGGAACUCGCUAUAAGUGCAGU
siNdp52	CCCCAGUUCGGGAUAAAUAGUAG
siTBK1	AACUAAUCAGUGUUUCGAUAUCG
siGalectin-8	AUGGGUCCUGGACGAACCGUUGU
siNOD2	CAGAGAUGCCGACACCAUACUGG
siTRAF6	CAGGGUACAAUACGCCUUACAAU
siMyo6	AGTACCGACTTGTTCCCTATGG
siBeclin1	AGTACCGACTTGTTCCCTATGG
siLuc	AUCGUACGCGGAAUACUUCGAdTdT
check for Atg14L-F	AGCGTGTAAGCGATGAGGAG
check for Atg14L-R	TTGAACCAAGAGGTCACCGAG
check for p22phox-F	TCCACTTCCTGTTGTCGGTG
check for p22phox-R	CTTCTTTCGGACCTCTGCGG
check for TLR2-F	CTTGGTTCTTTTCCCAAACTGGAG
check for TLR2-R	AGGCGGAGAGTCACACAGG
check for TBK1-F	GTCGGAAGAGTGGATGAGAAAG
check for TBK1-R	GAGCATTTTCTGAGGCAGAGT
check for Galectin-8-F	ATACAAAAGCCAGGCAAGCTCC
check for Galectin-8-R	GGTCAACATTAAAGCTTCGGGC
check for NOD2-F	CACCTGAACAGAGATGCCGA
check for NOD2-R	GACACGGTCAGGATGGTCAA
check for TRAF6-F	ACAGCAGTGTAACGGGATCT
check for TRAF6-R	CGGGTAGAGACTTCACAGCG
check for Myo6-F	ATGCAAGGGGGTTTCCCTTC
check for Myo6-R	GCGACTACAGACTAGCCACA
check for Beclin1-F	CGGACAGTTTGGCACAATCA
check for Beclin1-R	GTTGTCCCAGAAAAACCGCA
check for beta Actin-F	TTGCTGACAGGATGCAGAAG
check for beta Actin-R	ACATCTGCTGGAAGGTGGAC
si human Atg16L1	AGACTTTGCTGCGTAGATCCC
si human FIP200	GCCTAGAACAACTAACGAA
check for human FIP200-F	CTGCAATCATGGCCAACCTG
check for human FIP200-R	TCAGCATCTGCGGTATCTGG

Supplementary Table 3. Sequences of Primers for *ply* Gene Substitution in *S. pneumoniae*.

ply R6-N-F	GCCGATAAGGAAAAGATGAGCG
ply 5 rep-N-R anti	GTCATTTACTGCTTTATTTGCCAT
ply 5 rep-F	ATGGCAAATAAAGCAGTAAATGAC
ply 5 rep-R with *erm*	CGTACGCTAGCAATTGTACACTAATCATTTTCTACCTTATC
erm+ *ply* R6-C-F	AATGGACTAATGAAAATGTAAATTTAACTAGAGAGGAGAATGCTTGCGAC
ply R6-C-R anti	GAGTTGGCTCCATCTTTAGC
ply R6-R with *erm*	CGTACGCTAGCAATTGTACACTAGTCATTTTCTACCTTATC
erm-F	TGTACAATTGCTAGCGTACG
erm-R	TAGTTAAATTTACATTTTCATTAGTCCATT
