# Supplementary material for: Streptococcus pneumoniae triggers hierarchical autophagy through reprogramming of LAPosome-like vesicles via NDP52-delocalization
Source: Commun Biol. 2020 Jan 13;3:25. doi: 10.1038/s42003-020-0753-3 (PMC6957511; doi:10.1038/s42003-020-0753-3)
Supplement: Supplementary file 2 — Description of additional supplementary files [file 42003_2020_753_MOESM2_ESM.docx]

**Description of additional supplementary files**

**Supplementary Data 1.** Source data used to plot as follows:

Figure 1B, D, E, F, G, H, I, L, M

Figure 2B, C, E, F, G, G, H, I, J, K, L, M

Figure 3A, B, C, E, F, G, H, I

Figure 4A, B, C, D, E, F, G, H

Figure 5A, B, C, D, E, F, G

Figure 6I
