# Supplementary material for: Streptococcus pneumoniae triggers hierarchical autophagy through reprogramming of LAPosome-like vesicles via NDP52-delocalization
Source: Commun Biol. 2020 Jan 13;3:25. doi: 10.1038/s42003-020-0753-3 (PMC6957511; doi:10.1038/s42003-020-0753-3)
Supplement: Supplementary file 4 — Reporting summary [file 42003_2020_753_MOESM4_ESM.pdf]

nature research

Corresponding author(s): MICHINAGA OGAWA

Last updated by author(s): 2019-Nov-29

Reporting Summary

Nature Research wishes to improve the reproducibility of the work that we publish. This form provides structure for consistency and transparency in reporting. For further information on Nature Research policies, see Authors & Referees and the Editorial Policy Checklist .

Statistics

For all statistical analyses, confirm that the following items are present in the figure legend, table legend, main text, or Methods section.

n/a	Confirmed	
[ ]	[x]	The exact sample size (*n*) for each experimental group/condition, given as a discrete number and unit of measurement
[ ]	[x]	A statement on whether measurements were taken from distinct samples or whether the same sample was measured repeatedly
[ ]	[x]	The statistical test(s) used AND whether they are one- or two-sided<br>*Only common tests should be described solely by name; describe more complex techniques in the Methods section.*
[x]	[ ]	A description of all covariates tested
[ ]	[x]	A description of any assumptions or corrections, such as tests of normality and adjustment for multiple comparisons
[ ]	[x]	A full description of the statistical parameters including central tendency (e.g. means) or other basic estimates (e.g. regression coefficient) AND variation (e.g. standard deviation) or associated estimates of uncertainty (e.g. confidence intervals)
[ ]	[x]	For null hypothesis testing, the test statistic (e.g. *F*, *t*, *r*) with confidence intervals, effect sizes, degrees of freedom and *P* value noted<br>*Give P values as exact values whenever suitable.*
[x]	[ ]	For Bayesian analysis, information on the choice of priors and Markov chain Monte Carlo settings
[x]	[ ]	For hierarchical and complex designs, identification of the appropriate level for tests and full reporting of outcomes
[x]	[ ]	Estimates of effect sizes (e.g. Cohen's *d*, Pearson's *r*), indicating how they were calculated

Our web collection on statistics for biologists contains articles on many of the points above.

Software and code

Policy information about availability of computer code

Data collection	No custom software was used.
Data analysis	Statistical analyses were performed by using GraphPad Prism 6.0.

For manuscripts utilizing custom algorithms or software that are central to the research but not yet described in published literature, software must be made available to editors/reviewers. We strongly encourage code deposition in a community repository (e.g. GitHub). See the Nature Research guidelines for submitting code & software for further information.

Data

Policy information about availability of data

All manuscripts must include a data availability statement. This statement should provide the following information, where applicable:

- Accession codes, unique identifiers, or web links for publicly available datasets
- A list of figures that have associated raw data
- A description of any restrictions on data availability

The authors declare that all data supporting the findings reported in this study are available within the paper and its supplementary information files.

Field-specific reporting

Please select the one below that is the best fit for your research. If you are not sure, read the appropriate sections before making your selection.

[x] Life sciences [ ] Behavioural & social sciences [ ] Ecological, evolutionary & environmental sciences

For a reference copy of the document with all sections, see nature.com/documents/nr-reporting-summary-flat.pdf

Life sciences study design

All studies must disclose on these points even when the disclosure is negative.

Sample size	At least independent triplicates were performed in all experiments. Sample sizes were determined based on the generating a convincing result.
Data exclusions	No data exclusions were needed.
Replication	At least 500 PcV, PcLV, PcAV, or each marker-positive PcVcontaining cells were examined in triplicate . All attempts at replication were successful.
Randomization	Samples were allocated randomly into experimental groups.
Blinding	The investigator was blinded during data collection and analysis.

Reporting for specific materials, systems and methods

We require information from authors about some types of materials, experimental systems and methods used in many studies. Here, indicate whether each material, system or method listed is relevant to your study. If you are not sure if a list item applies to your research, read the appropriate section before selecting a response.

Materials & experimental systems

n/a	Involved in the study	
☐	☒	Antibodies
☐	☒	Eukaryotic cell lines
☒	☐	Palaeontology
☒	☐	Animals and other organisms
☒	☐	Human research participants
☒	☐	Clinical data

Methods

n/a	Involved in the study	
☒	☐	ChIP-seq
☒	☐	Flow cytometry
☒	☐	MRI-based neuroimaging

Antibodies

Antibodies used	Anti-S. pneumoniae (SSI), anti-GFP (Cell Signaling), anti-Myc (9B11, Cell Signaling), anti-Flag (Wako), anti-Galectin-3 (SINO BIOLOGICAL), anti-Calcoco2 (Proteintech for WB), anti-NDP52 (Gene Tex (GTX115378) for IF), anti-K63 linked Ub (clone Apu3, EMD Millipore), anti-LC3, p62, RFP, Atg16L1, ubiquitin (MBL), and anti-actin (Santa Cruz Biotechnology, Inc.) were used as primary antibodies. An HRP-conjugated goat anti-rabbit or anti-mouse antibodies (Jackson Laboratories) were used as secondary antibodies for immunoblotting. FITC- or TRITC-conjugated goat anti-rabbit or anti-mouse IgG antibodies (Sigma-Aldrich) were used as secondary antibodies for immunostaining.
Validation	Antibodies have been validated by the manufacturers and previously used for the same applications (Ogawa et al., Cell Microbiol, 20, e12846 (2018))

Eukaryotic cell lines

Policy information about cell lines

Cell line source(s)	293T, platE, and A549. FIP200 WT or KO, ULK1/2 WT or DKO (double knockout) , Atg5 WT or KO, Nox1 WT or KO and Nox4 WT or KO , Atg16L1 WT or KO, and p62 WT or KO MEFs were generated and validated by Dr.Jun-Lin Guan (University of Cincinnati College of Medicine), Dr. Craig B. Thompson (Memorial Sloan Kettering Cancer Center), Dr. Noboru Mizushima (The University of Tokyo), Dr. Denis Martinvalet (Genève University), and Drs. Tatsuya Saitoh (Tokushima University) and Shizuo Akira (Osaka University), and Toru Yanagawa (University of Tsukuba).
Authentication	Authenticated by ATCC, manufacturer, and suppliers.
Mycoplasma contamination	All cell lines were negative in Mycoplasma contamination test.
Commonly misidentified lines (See ICLAC register)	There was no misidentified cell lines in this study.
